# Putative EPHX1 Enzyme Activity Is Related with Risk of Lung and Upper Aerodigestive Tract Cancers: A Comprehensive Meta-Analysis

**DOI:** 10.1371/journal.pone.0014749

**Published:** 2011-03-18

**Authors:** Xiang Li, Zheng Hu, Xinshun Qu, Jiadong Zhu, Lin Li, Brian Z. Ring, Li Su

**Affiliations:** 1 Key Laboratory of Molecular Biophysics of Ministry of Education, School of Life Science and Technology, Sino-France Joint Center for Drug Research and Screening, Huazhong University of Science and Technology, Wuhan, China; 2 Beijing Institute of Genomics, Chinese Academy of Sciences, Beijing, China; 3 Department of Plant Pathology, The Pennsylvania State University, University Park, Pennsylvania, United States of America; 4 Yigene Inc, Jianwai, Beijing, China; Université de Montréal, Canada

## Abstract

**Background:**

EPHX1 is a key enzyme in metabolizing some exogenous carcinogens such as products of cigarette-smoking. Two functional polymorphisms in the *EPHX1* gene, Tyr113His and His139Arg can alter the enzyme activity, suggesting their possible association with carcinogenesis risk, particularly of some tobacco-related cancers.

**Methodology/Principal Findings:**

A comprehensive systematic review and meta-analysis was performed of available studies on these two polymorphisms and cancer risk published up to November 2010, consisting of 84 studies (31144 cases and 42439 controls) for Tyr113His and 77 studies (28496 cases and 38506 controls) for His139Arg primarily focused on lung cancer, upper aerodigestive tract (UADT) cancers (including oral, pharynx, larynx and esophagus cancers), colorectal cancer or adenoma, bladder cancer and breast cancer. Results showed that Y113H low activity allele (H) was significantly associated with decreased risk of lung cancer (OR = 0.88, 95%CI = 0.80–0.96) and UADT cancers (OR = 0.86, 95%CI = 0.77–0.97) and H139R high activity allele (R) with increased risk of lung cancer (OR = 1.18, 95%CI = 1.04–1.33) but not of UADT cancers (OR = 1.05, 95%CI = 0.93–1.17). Pooled analysis of lung and UADT cancers revealed that low EPHX1 enzyme activity, predicted by the combination of Y113H and H139R showed decreased risk of these cancers (OR = 0.83, 95%CI = 0.75–0.93) whereas high EPHX1 activity increased risk of the cancers (OR = 1.20, 95%CI = 0.98–1.46). Furthermore, modest difference for the risk of lung and UADT cancers was found between cigarette smokers and nonsmokers both in single SNP analyses (low activity allele H: OR = 0.77/0.85 for smokers/nonsmokers; high activity allele R: OR = 1.20/1.09 for smokers/nonsmokers) and in combined double SNP analyses (putative low activity: OR = 0.73/0.88 for smokers/nonsmokers; putative high activity: OR = 1.02/0.93 for smokers/ nonsmokers).

**Conclusions/Significance:**

Putative low EPHX1 enzyme activity may have a potential protective effect on tobacco-related carcinogenesis of lung and UADT cancers, whereas putative high EPHX1 activity may have a harmful effect. Moreover, cigarette-smoking status may influence the association of EPHX1 enzyme activity and the related cancer risk.

## Introduction

Human microsomal epoxide hydrolase (EPHX1 or mEH, EC 3.3.2.9) plays an important role during xenobiotic detoxification of exogenous chemicals such as polycyclic aromatic hydrocarbons (PAHs) which are produced during the use of coal tar, coke, bitumen, or during cigarette smoking [1]–[3]. On the other hand, it is also involved in the xenobiotic activation of some carcinogens [4]–[6]. EPHX1 also hydrolyzes arene, alkene, and aliphatic epoxides, which are metabolic products from PAHs and aromatic amines by cytochrome P450 and other phase I enzymes catalysis [1].

The human *EPHX1* gene is 35.48 kb with nine exons and eight introns on chromosome 1q42.1. To date, more than 110 single nucleotide polymorphisms (SNPs) have been identified according to the NCBI's dbSNP database. Two SNPs among them, Tyr113His (rs1051740, in exon 3) and His139Arg (rs2234922, in exon 4), have been well characterized both *in vitro* studies and epidemiological investigation. Early *in vitro* studies showed that EPHX1 enzymatic activity was decreased by approximately 40% in subjects with the His113 allele (low EPHX1 activity allele) and increased by at least 25% with the Arg139 allele (high EPHX1 activity allele) [7], [8]. Given the known differential effect of *EPHX1* alleles in the detoxification of procarcinogens, it has been proposed that these polymorphisms may affect cancer risk. Later population studies found that these two functional polymorphisms were strongly associated with susceptibility to a number of cancers, such as lung cancer [9]–[12], upper aerodigestive tract (UADT) cancers [13], [14], colorectal cancer or adenoma [15], [16], bladder cancer [17], breast cancer [18]. Based on the genotype combination of these two functional polymorphisms, Benhamou and colleagues [9] classified EPHX1 activity as putative low activity (113HH/139HH, 113HH/139HR and 113YH/139HH), intermediate activity (113HH/139RR, 113YY/139HH and 113YH/139HR) and high activity (113YH/139RR, 113YY/139HR and 113YY/139RR). They also found a significant association with lung cancer risk between cases exhibiting putative high and intermediate EPHX1 activity compared to low activity cases in Caucasian cigarette smokers [9]. A previous meta-analysis of the association of these SNPs with lung cancer revealed that the low-activity genotype (HH) of *EPHX1* polymorphism Y113H was associated with decreased risk of lung cancer while the high-activity genotype (RR) of polymorphism H139R was associated with a modest increase risk of lung cancer among Caucasians. Moreover, the predicted low activity by genotype combination of two polymorphisms was associated with a modest decrease of lung cancer risk [19]. However, it has not been well clarified whether EPHX1 enzymatic activity is associated with cancer risk.

The present comprehensive meta-analysis of published epidemiological studies aims to systematically evaluate putative EPHX1 enzyme activity and risk of cancers predicted by single polymorphism of Y113H/H139R and by combined double polymorphisms, and to identify the association between these two functional polymorphisms and risk of some tobacco-related cancers.

## Materials and Methods

### Search strategy

All case-control studies of EPHX1 polymorphisms and cancer risk published up to November 1, 2010 were identified through comprehensive searches in PubMed, EMBASE, ISI Web of Science and Google Scholar. The search terms used were: *EPHX1, microsomal epoxide hydrolase and mEH* in combination with *polymorphism, variation, genotype, genetic and mutation*, and in combination with *cancer, tumor, tumour, carcinoma, adenoma and adenocarcinoma*. For each identified study, additional studies were sought from its references, citations and from the PubMed option ‘Related Articles’.

### Selection

The following criteria were employed to determine inclusion of a study in this meta-analysis: 1) a case–control study evaluating at least one of these two polymorphisms (Y113H and H139R) and cancer risk; 2) no overlapping data. All data were independent of each other. For the same or overlapping data in the studies published by the same researchers, we selected the most recent study with a larger number of participants; 3) full-text articles; 4) published in English language journals.

### Data Extraction

The collected data items included: first author, published year, cancer type, study design, original country, sample ethnicity, sample size, genotype counts and genotyping method. The data were independently extracted by two investigators (Li and Zhu) and rechecked by Hu. All item-specific ambiguities were clarified by investigators' consultation. Different case-control groups in one study were considered as independent studies. Cigarette smoking status was strategically classified as current smokers and nonsmokers.

### Quantitative data synthesis

To evaluate the association of EPHX1 polymorphisms with carcinogenesis risk, we treated wild-type Y of Y113H and H of H139R as intermediate activity alleles and treated wild-type YY of Y113H and HH of H139R as intermediate activity genotypes. They are the comparison references for calculating odds ratios. Thus comparisons are Low activity vs. Intermediate activity (H vs. Y, YH vs. YY and HH vs. YY) and High activity vs. Intermediate activity (R vs. H, HR vs. HH and RR vs. HH) (Table S1).

EPHX1 enzymatic activity was also predicted by double polymorphisms based on the method of Benhamou 1998 [9] namely low activity (113HH/139HH, 113HH/139HR and 113YH/139HH), intermediate activity (113HH/139RR, 113YY/139HH and 113YH/139HR) and high activity (113YH/139RR, 113YY/139HR and 113YY/139RR) (Table S1).

Random-effects methods [20] were used to calculate pooled odds ratios (ORs) and the associated 95% confidence intervals (CIs).

The Cochran's Q statistic [21] and the inconsistency index *I^2^* [22] were used to evaluate the between-study heterogeneity. Random effect meta-regression models with restricted maximum likelihood estimation were employed to evaluate the different variance among the individual ORs when heterogeneity was detected. The pre-specified possible sources of inter-study heterogeneity were: cancer type, ethnicity of population (Caucasian, East Asian, South Asian, African or Mixed population), study design (hospital-based case-control study, population-based case-control study or nested case-control study), sample size (≥500 or <500) and HWE violation (violated or not violated). Furthermore, the sensitive analysis method proposed by Patsopoulos et al. was implemented to identify studies which may be the main source of the measured heterogeneity [23].

To detect potential publication bias, funnel plots [24] were applied by plotting individual study log OR against the standard error of the log OR. Plots should resemble a symmetrical inverted funnel if ascertainment bias was absent. Publication bias was also assessed using Egger's test [25], by which asymmetry in a funnel plot could be tested.

Except for heterogeneity statistics (where significance was declared if *P*-value < 0.10), all results were considered “significant” if the corresponding *P*-value was < 0.05. All *P-*values were 2-sided. The statistical analyses were performed using STATA 11.0 (STATA Corp, College Station, Texas).

## Results

### Flow of included studies

**Figure 1:**
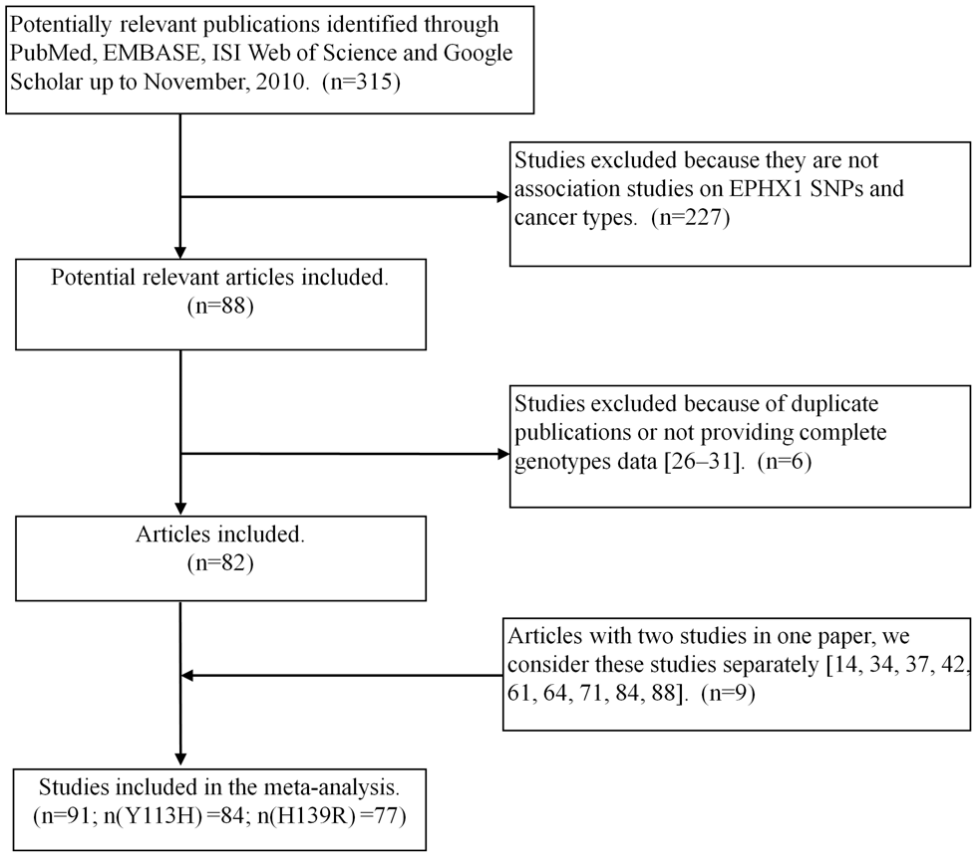
Flow chart of study selection.

Initially a total of 315 potentially relevant publications up to November 1, 2010 were identified through PubMed, EMBASE, ISI Web of Science and Google Scholar. 227 studies were excluded because of insufficient information related to pre-specified inclusion criteria. Further six studies were excluded because of a duplicated publication or for not providing complete genotypes data [26]–[31]. The reasons for exclusion of each case-control study were detailed in Text S1. Finally, 82 articles [9]–[18], [32]– were selected in the meta-analysis, of which 9 articles [14], [34], [37], [42], [61], [64], [71], [84], [88] had two independent studies and were considered separately. Therefore, a total of 91 studies, of which 84 studies (31144 cases and 42439 controls) for Tyr113His and 77 studies (28496 cases and 38506 controls) for His139Arg dominated by lung cancer, UADT, colorectal cancer, colorectal adenoma, bladder cancer, breast cancer, liver cancer and blood cancers (leukemia, lymphoma and multiple myeloma) were included in the meta-analysis based on our search strategy and eligibility criteria (Table S2 and Figure 1).

### Study characteristics

Detailed characteristics of the aggregated data for 91 case-control studies are listed in Table S2. Minor allele frequency of Y113H and H139R of controls in different populations graphed as Figure S1. Among overall studies, 24 studies (6418 cases and 9516 controls) that further evaluated the putative EPHX1 enzyme activity and cancer risk by the method described by Benhamou et al. [9] are characterized in Table S3.

### Quantitative data synthesis

#### EPHX1 polymorphisms Y113H and H139R and cancer risk

The associations of each of *EPHX1* Y113H and H139R polymorphisms with cancer risk were analyzed. Summary ORs for cancer risk of *EPHX1* Y113H and H139R polymorphisms in different cancer types were shown in Table 1. The overall OR by the random-effects model showed no significant association between Y113H or H139R and cancer risk except between the heterozygote versus wild-type Y113H allele (YH vs. YY), which exhibited a slightly decreased cancer risk (OR = 0.94, 95%CI = 0.90–0.99; *P = *0.016). Results of analyzing these two polymorphisms in different cancer types revealed that the low activity allele (H) of Y113H was highly associated with decreased risk of lung cancer (OR = 0.88, 95%CI = 0.80–0.96; *P = *0.005) and UADT (OR = 0.86, 95%CI = 0.77–0.97; *P = *0.014); the high activity allele (R) of H139R was significantly associated with increased risk of lung cancer (OR = 1.18, 95%CI = 1.04–1.33; *P = *0.010) but not of UADT (OR = 1.05, 95%CI = 0.93–1.17, *P = *0.447). However, the homozygous variant (RR) of H139R showed increased risk of UADT cancers (OR = 1.34, 95%CI = 0.98–1.82, *P = *0.065). Towards other assessed cancers, i.e., colorectal cancer, colorectal adenoma, breast cancer, bladder cancer, blood cancers (leukemia, lymphoma and multiple myeloma) or liver cancer, the study revealed only modest decreased or increased effects on cancer risk: Y113H for blood cancers (YH vs. YY: OR = 0.78, 95%CI = 0.60–1.00) and H139R for colorectal cancer (HR vs. HH: OR = 0.91, 95%CI = 0.84–1.00). No statistically significant association was observed for each polymorphism with other cancer cases. Interestingly, though not significant, the low activity of Y113H showed increased risk of bladder cancer (H vs. Y: OR = 1.17, 95%CI = 0.92–1.49; HH vs. YY: OR = 1.27, 95%CI = 0.84–1.92) whereas high activity of H139R showed decreased risk of bladder cancer (R vs. H: OR = 0.89, 95%CI = 0.76–1.05; RR vs. HH: OR = 0.73, 95%CI = 0.84–1.92) (Table 1).

**Table 1:** Summary ORs for association of *EPHX1* polymorphisms Y113H and H139R with different cancers.

		Y113H					H139R			
Cancer group	Low vs.	Random effects	*P*-value	*P*-value for	*I^2^*	High vs.	Random effects	*P*-value	*P*-value for	*I^2^*
(Studies and cases/controls)	Intermediate	OR (95% CI)		heterogeneity		Intermediate	OR (95% CI)		heterogeneity	
**Overall**	(N = 84; 31144/42439)					(N = 77; 28496/38506)				
	H vs. Y	0.99 (0.95–1.03)	0.574	<0.001	61.4%	R vs. H	1.02 (0.98–1.06)	0.470	0.002	35.5%
	YH vs. YY	0.94 (0.90–0.99)	0.016	<0.001	43.2%	HR vs. HH	1.00 (0.95–1.04)	0.868	0.011	29.1%
	HH vs. YY	1.02 (0.94–1.12)	0.592	<0.001	59.2%	RR vs. HH	1.08 (0.98–1.20)	0.141	0.043	23.0%
**Lung cancer**	(N = 18; 4819/9049)					(N = 18; 6742/9151)				
	H vs. Y	0.88 (0.80–0.96)	0.005	0.033	41.7%	R vs. H	1.18 (1.04–1.33)	0.010	< 0.001	68.3%
	YH vs. YY	0.86 (0.78–0.95)	0.003	0.272	15.2%	HR vs. HH	1.19 (1.04–1.36)	0.012	0.001	59.4%
	HH vs. YY	0.81 (0.65–1.00)	0.048	0.037	41.9%	RR vs. HH	1.22 (0.92–1.63)	0.162	0.018	45.7%
**UADT cancers**	(N = 15; 3285/5324)					(N = 14; 2963/4867)				
	H vs. Y	0.86 (0.77–0.97)	0.014	0.002	58.8%	R vs. H	1.05 (0.93–1.17)	0.447	0.110	33.1%
	YH vs. YY	0.77 (0.66–0.90)	0.001	0.027	45.9%	HR vs. HH	1.00 (0.87–1.17)	0.874	0.138	29.9%
	HH vs. YY	0.82 (0.66–1.03)	0.084	0.010	51.8%	RR vs. HH	1.34 (0.98–1.82)	0.065	0.252	18.5%
**Colorectal cancer**	(N = 11; 5283/6903)					(N = 10; 4456/5669)				
	H vs. Y	1.04 (0.96–1.13)	0.310	0.089	40.3%	R vs. H	0.95 (0.88–1.02)	0.144	0.857	0.0%
	YH vs. YY	1.05 (0.97–1.14)	0.199	0.820	0.0%	HR vs. HH	0.91 (0.84–1.00)	0.041	0.806	0.0%
	HH vs. YY	1.11 (0.89–1.39)	0.351	0.005	61.8%	RR vs. HH	1.01 (0.82–1.26)	0.897	0.545	0.0%
**Colorectal adenoma**	(N = 8; 4012/4057)					(N = 9; 4857/4929)				
	H vs. Y	0.95 (0.88–1.03)	0.224	0.259	21.4%	R vs. H	1.05 (0.98–1.13)	0.193	0.490	0.0%
	YH vs. YY	0.94 (0.86–1.04)	0.239	0.510	0.0%	HR vs. HH	1.04 (0.95–1.13)	0.418	0.696	0.0%
	HH vs. YY	0.92 (0.78–1.09)	0.319	0.212	27.1%	RR vs. HH	1.13 (0.92–1.38)	0.233	0.460	0.0%
**Breast cancer**	(N = 6; 6090/7797)					(N = 4; 4543/6899)				
	H vs. Y	0.98 (0.90–1.07)	0.696	0.123	42.3%	R vs. H	0.96 (0.89–1.02)	0.200	0.835	0.0%
	YH vs. YY	0.97 (0.90–1.04)	0.411	0.618	0.0%	HR vs. HH	0.95 (0.87–1.03)	0.202	0.749	0.0%
	HH vs. YY	1.06 (0.81–1.39)	0.680	0.002	72.8%	RR vs. HH	0.94 (0.78–1.15)	0.557	0.634	0.0%
**Bladder cancer**	(N = 5; 1810/1869)					(N = 4; 1614/1656)				
	H vs. Y	1.17 (0.92–1.49)	0.192	0.002	76.6%	R vs. H	0.89 (0.76–1.05)	0.168	0.262	25.0%
	YH vs. YY	1.25 (0.90–1.75)	0.183	0.006	72.1%	HR vs. HH	0.91 (0.78–1.06)	0.240	0.610	0.0%
	HH vs. YY	1.27 (0.84–1.92)	0.266	0.015	67.4%	RR vs. HH	0.73 (0.40–1.32)	0.300	0.155	42.8%
**Blood cancers**	(N = 7; 1844/2028)					(N = 10; 2217/3067)				
	H vs. Y	0.98 (0.86–1.11)	0.743	0.255	22.8%	R vs. H	0.95 (0.86–1.05)	0.318	0.426	1.3%
	YH vs. YY	0.78 (0.60–1.00)	0.046	0.059	50.6%	HR vs. HH	0.96 (0.82–1.12)	0.607	0.241	22.0%
	HH vs. YY	1.08 (0.85–1.37)	0.513	0.329	13.2%	RR vs. HH	0.96 (0.62–1.48)	0.838	0.136	35.2%
**Liver cancer**	(N = 4; 368/859)					(N = 3; 212/556)				
	H vs. Y	1.05 (0.74–1.48)	0.790	0.036	65.0%	R vs. H	1.11 (0.80–1.54)	0.537	0.222	33.6%
	YH vs. YY	0.90 (0.59–1.39)	0.639	0.087	54.4%	HR vs. HH	1.18 (0.83–1.68)	0.366	0.738	0.0%
	HH vs. YY	1.16 (0.57–2.40)	0.681	0.049	61.8%	RR vs. HH	1.05 (0.34–3.29)	0.927	0.116	53.6%

UADT, upper aerodigestive tract; N, number of studies.

**Figure 2:**
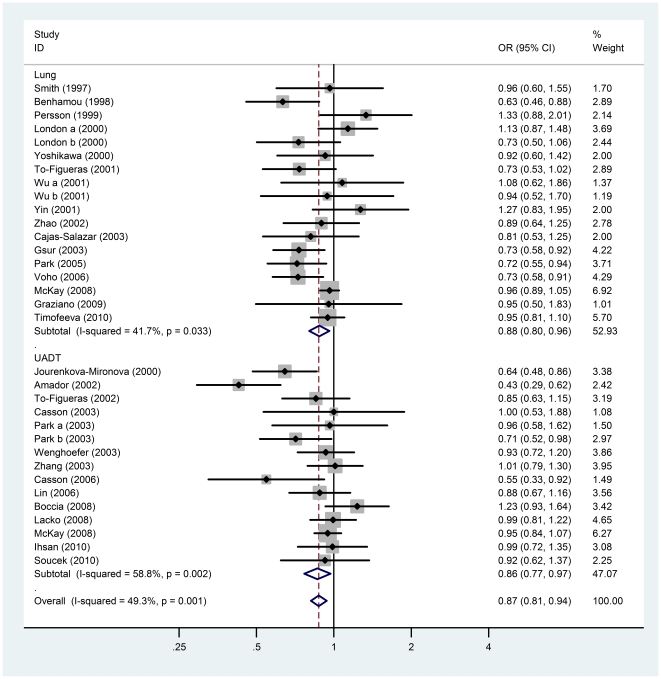
Forest plots describing the association of *EPHX1* polymorphism Y113H with lung and upper aerodigestive tract (UADT) cancers. ORs were calculated by comparing the low activity allele H vs. the intermediate activity allele Y in lung and UADT cancers. *P*-values of the ORs are calculated with the DerSimonian-Laird method using a random effects model and measurements of heterogeneity are based on Cochran's Q-test and the inconsistency index *I* ^2^.

As lung and UADT cancers share a similar etiology and association with tobacco usage [104], we pooled lung and UADT cancers together to further explore the cancer risk of polymorphisms Y113H and H139R. We found that the low activity allele (H) of Y113H presented a significant association with decreased cancer risk (OR = 0.87, 95%CI = 0.81–0.94; *P = *0.0002) (Table 2 and Figure 2), whereas the high activity allele (R) of H139R presented a modest association with increased cancer risk (OR = 1.12, 95%CI = 1.03–1.22; *P = *0.011) (Table 2).

**Table 2:** Summary ORs for association of *EPHX1* polymorphisms Y113H and H139R with pooled lung and upper aerodigestive tract (UADT) cancers.

		Y113H					H139R			
Study group	N	Random effects OR (95% CI)	*P*-value	*P*-value for	*I^2^*	N	Random effects OR (95% CI)	*P*-value	*P*-value for	*I^2^*
		Low vs. Intermediate (H vs. Y)		heterogeneity			High vs. Intermediate (R vs. H)		heterogeneity	
**Overall**										
Lung + UADT	33	0.87 (0.81–0.94)	0.0002	0.001	49.3%	32	1.12 (1.03–1.22)	0.011	<0.001	58.0%
**Ethnicity**										
Caucasian	21	0.87 (0.81–0.94)	0.0005	0.015	44.5%	24	1.07 (0.97–1.15)	0.228	0.001	58.7%
Asian	6	1.02 (0.89–1.16)	0.806	0.553	0.0%	3	1.52 (1.13–2.05)	0.006	0.438	0.0%
African	3	0.83 (0.63–1.09)	0.175	0.619	0.0%	3	1.26 (1.01–1.57)	0.040	0.741	0.0%
**Study design**										
Population-based	21	0.88 (0.80–0.96)	0.006	0.026	41.3%	18	1.15 (1.01–1.31)	0.033	<0.001	64.2%
Hospital-based	9	0.82 (0.68–0.98)	0.031	0.004	64.3%	10	1.09 (0.96–1.25)	0.187	0.310	14.5%
**Sample size**										
≥500	10	0.91 (0.84–0.98)	0.022	0.061	44.7%	10	1.02 (0.92–1.13)	0.695	0.001	67.6%
<500	23	0.85 (0.76–0.95)	0.004	0.005	48.5%	22	1.21 (1.07–1.37)	0.002	0.038	38.0%
**Smoke status** *										
Nonsmokers	7	0.85 (0.69–1.06)	0.152	0.942	0.0%	4	1.09 (0.70–1.68)	0.709	0.130	46.9%
Smokers	7	0.77 (0.65–0.91)	0.002	0.664	0.0%	7	1.20 (0.93–1.55)	0.152	0.119	40.8%

N, number of studies; * YH+HH vs. YY for Y113H; HR+RR vs. HH for H139R.

The ethnicity, study design, sample size and smoke status of pooled lung and UADT cancer risk carrying the low enzymatic activity allele (H) of Y113H or the high enzymatic activity allele (R) of H139R was calculated and a modest difference between cigarette smokers and nonsmokers was observed (Table 2). The odds ratio for the low activity allele (H) of polymorphism Y113H in smokers was 0.77 (95%CI = 0.65–0.91, *P = *0.002) and 0.85 (95%CI = 0.69–1.06, *P = *0.152) in nonsmokers. The odds ratio for high activity allele (R) of polymorphism H139R was 1.20 (95%CI = 0.93–1.55, *P = *0.152) in smokers and 1.09 (95%CI = 0.70–1.68, *P = *0.709) in nonsmokers.

#### Putative EPHX1 enzyme activity and risk of lung and UADT cancers

**Figure 3:**
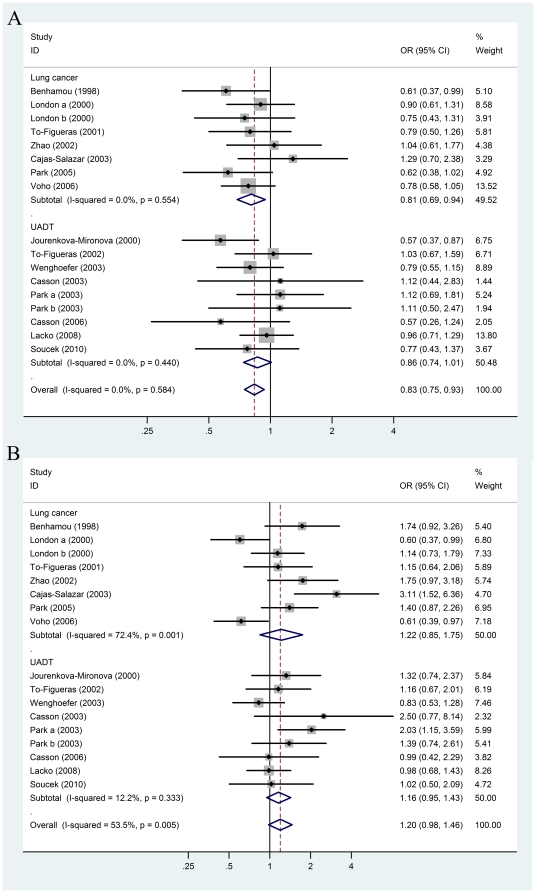
Forest plots describing the association of putative *EPHX1* enzyme activities with lung and upper aerodigestive tract (UADT) cancers. ORs were calculated as (**A**) putative low activity vs. putative intermediate activity, and (**B**) putative high activity vs. putative intermediate activity predicted by genotype combination of polymorphisms Y113H/H139R in lung and UADT cancers. *P*-values of the ORs are calculated with the DerSimonian-Laird method using a random effects model and measurements of heterogeneity are based on Cochran's Q-test and the inconsistency index *I* ^2^.

In order to evaluate the association of these two functional polymorphisms and their enzyme activity with carcinogenesis risk, we analyzed the association of EPHX1 enzyme activity predicted by genotype combination of polymorphism Y113H and H139R with risk of lung and UADT cancers. In an overall comparison to the putative intermediate EPHX1 activity, low EPHX1 activity decreased risk of lung and UADT cancers significantly (OR = 0.83; 95%CI = 0.75–0.93, *P = *0.001) and high EPHX1 activity increased the cancer risk (OR = 1.20; 95%CI = 0.98–1.46; *P = *0.081) (Table 3 and Figure 3).

**Table 3:** Summary ORs for association of putative *EPHX1* enzyme activity by Y113H/H139R genotype combination with lung and upper aerodigestive tract (UADT) cancers.

		Low vs. Intermediate			High vs. Intermediate		
Study group	N (cases/controls)	Random effects	*P* value	*P*-value for	*I^2^*	Random effects	*P* value	*P*-value for	*I^2^*
		OR (95%CI)		heterogeneity		OR (95%CI)		heterogeneity	
**Overall**									
Lung + UADT	17 (2928/5436)	0.83 (0.75–0.93)	0.001	0.584	0.0%	1.20 (0.98–1.46)	0.081	0.005	53.5%
**Ethnicity**									
Caucasian	15 (2692/5072)	0.83 (0.74–0.94)	0.002	0.483	0.0%	1.20 (0.95–1.51)	0.125	0.002	58.9%
African	2 (236/364)	0.85 (0.54–1.35)	0.497	0.427	0.0%	1.22 (0.86–1.76)	0.287	0.625	0.0%
**Study design**									
Hospital-based	6 (959/1063)	0.75 (0.59–0.95)	0.016	0.273	21.3%	1.30 (0.96–1.76)	0.087	0.177	34.6%
Population-based	11 (1969/4373)	0.88 (0.77–1.00)	0.050	0.799	0.0%	1.15 (0.88–1.50)	0.303	0.004	60.9%
**Sample size**									
≥500	5 (1296/3606)	0.83 (0.71–0.97)	0.020	0.633	0.0%	0.85 (0.63–1.13)	0.263	0.074	53.1%
<500	12(1632/1830)	0.84 (0.70–0.99)	0.036	0.393	5.3%	1.44 (1.20–1.73)	0.0001	0.411	3.5%
**Smoke status**									
Nonsmokers	2 (191/1719)	0.88 (0.61–1.28)	0.517	0.535	0.0%	0.93 (0.58–1.51)	0.777	0.866	0.0%
Smokers	4 (620/1132)	0.73 (0.58–0.93)	0.009	0.825	0.0%	1.02 (0.68–1.53)	0.928	0.136	46.0%

The association of EPHX1 enzyme activity predicted by the combination of these two polymorphisms with the risk of cigarette smoker or nonsmoker in pooled lung and UADT cancers was further assessed. Similar results with the single SNP analysis were obtained: modest, non-significant differences of ORs of putative EPHX1 enzyme activity between cigarette smokers and nonsmokers. The odds ratio of putative low enzyme activity was 0.73 (95%CI = 0.58–0.93; *P = *0.009) in smokers and 0.88 (95%CI = 0.61–1.28) in nonsmokers while the odds ratio of putative high enzyme activity was 1.02 (95%CI = 0.68–1.53) in smokers and the 0.93 (95%CI = 0.58–1.51) in nonsmokers (Table 3).

#### Between-study heterogeneity

Obvious Between-study heterogeneity was detected among pooled lung and UADT cancer studies on *I^2^* measures of 49.3% (*P* = 0.001) and 58.0% (*P*<0.001) for H vs. Y of Y113H and R vs. H of H139R, respectively. After subgroup analysis by ethnicity, we found heterogeneity was only obvious in Caucasian (H vs. Y of Y113H: *I^2^* = 44.5%, *P = *0.015; R vs. H of H139R: *I^2^* = 58.7%, *P = *0.001) but not in Asian and African descents (Table 2).

By further univariate meta-regression analysis, we identified that it is population ethnicity (β coefficient = 0.12 (0.03–0.21), *P* = 0.021) that was a significant source of heterogeneity for R vs. H of H139R but not cancer type, study design, sample size, genotyping method or HWE-violation (Table S4). Actually, it has shown that the high activity allele (R) of H139R significantly increased the cancer risk in Asians (OR = 1.52, 95%CI = 1.13–2.05) and Africans (OR = 1.26, 95%CI = 1.01–1.57) but not in Caucasians (OR = 1.07, 95%CI = 0.97–1.15) in pooled analysis. These results emphasized that the population heterogeneity of polymorphism H139R was associated with cancer risk. None of cancer type, ethnicity, study design, sample size, genotyping method or HWE-violation was found to be the source of heterogeneity for H vs. Y of Y113H (Table S4).

In the analysis of putative EPHX1 enzyme activity and risk of lung and UADT cancers, obvious between-study heterogeneity was identified for high vs. intermediate (*I^2^* = 53.5%, *P* = 0.005) (Table 3). Sample size was found the main source of heterogeneity (β coefficient = 0.53 (0.19–0.88), *P* = 0.005). When the studied samples were classified into large size subgroup (≥500, OR = 0.85, 95%CI = 0.63–1.13) and small size one (<500, OR = 1.44, 95%CI = 1.20–1.73), unexpectedly, it brought about the results that heterogeneity was still obvious in large sample subgroup (*I^2^* = 53.1%, *P* = 0.074) but not in small sample subgroup (*I^2^* = 3.5%, *P* = 0.411) (Table 3).

#### Sensitive analysis

Applying sensitive analysis method of Patsopoulos et al [23], we found studies of Jourenkova-Mironova [13], Benhamou [9] and Voho [41] contributed mostly to the heterogeneity in comparison of H vs. Y of Y113H in Caucasians. After excluded these three studies, the index *I^2^* decreased significantly from 44.5% (*P* = 0.015) to 18.5% (*P* = 0.232) and odds ratio became 0.92 (95%CI = 0.87–0.98). In comparison of R vs. H of H139R, studies of Zienolddiny [12] and Graziano [44] contributed the most to heterogeneity in Caucasians. After excluded them, the index *I^2^* decreased from 58.7% (*P* = 0.001) to 20.0% (*P* = 0.202) and odds ratio became 1.00 (95%CI = 0.94–1.06). These results indicated that the high activity allele (R) of H139R may be not associated with lung and UADT cancer risk in Caucasians when considering omitting heterogeneity-caused studies.

Three studies of Cajas-Salazar [40], Voho [41] and London a [34] were identified to contribute to the heterogeneity for high vs. intermediate of putative EPHX1 enzyme activity. After excluded them, the index *I^2^* decreased from 53.5% (*P* = 0.005) to 0.0% (*P* = 0.42) and odds ratio became 1.23 (95% = 1.06–1.42). Interestingly, the both studies of Cajas-Salazar [40] and Voho [41] were belong to the large sample subgroup (≥500). Therefore, though in large sample subgroup putative high activity showed decreased risk of lung and UADT caners, the studies were quite heterogeneous and the authentic role of EPHX1 high activity might increase risk of lung and UADT caners as data showed above.

#### Publication bias

By Begg's funnel plot and Egger's test, the results revealed a significant publication bias for H vs. Y of Y113H (*P* = 0.003) in the pooled analysis of lung and UADT cancers but not for R vs. H of H139R (*P = *0.141) (Figure S2). Among all studies, four studies of Zienolddiny [12], Ihsan [56], Graziano [44] and Wu a [37] were detected to deviate remarkably from other symmetrically distributed studies in the funnel plot (Figure S2). These four studies were exactly the source of heterogeneity from Patsopoulos et al's sensitive analysis in pooled dataset of lung and UADT cancers. When omitted these four studies, Egger's test *P*-value turned into 0.124, and *I^2^* decreased from 57.6% (P for heterogeneity <0.001) to 12.6% (P for heterogeneity <0.276).

## Discussion

The present meta-analysis provides the most comprehensive and up-to-date evidence on putative EPHX1 enzyme activity predicted from two genetic polymorphisms, Y113H and H139R, and risk of developing cancers. Though a number of early studies showed the two polymorphisms functionally affect the EPHX1 enzymatic activity [7], [8] and are associated with certain cancers [9]–[18], our systematic analyses revealed that both Y113H low enzymatic activity allele (H) and putative low EPHX1 enzyme activity, predicted by the combination of Y113H and H139R, were significantly associated with decreased risk of lung and UADT cancers, while the putative high EPHX1 enzyme activity was associated with increased risk of these cancers. Certainly, the actual EPHX1 enzyme activity should be measured in cancer case-control population to confirm cancer susceptibility of EPHX1 activity. Moreover, it showed modest difference of the risk of lung and UADT cancers between cigarette smokers and nonsmokers both in single SNP analyses and in combined double SNP analyses. Thus, cigarette-smoking status may influence the association of EPHX1 enzyme activity and the related cancer risk.

These findings are consistent with the known roles for EPHX1 enzyme in the detoxification and activation of exogenous carcinogens such as PAHs during tobacco smoking [3]. Lung and UADT cancers have been well characterized as causally related to cigarette smoking [104]. Smoking products such as tobacco-specific nitrosamines (4-(methylnitrosamino)-1-(3-pyridyl)-1-butanone, N’-nitrosonornicotine, etc.), polycyclic aromatic hydrocarbons (e.g. benzo[a]pyrene) and aromatic amines (e.g. 4-aminobiphenyl) are strongly toxic to epithelial cells and are potential carcinogens [105]. The EPHX1 enzyme has been proposed to transform epoxide intermediates from PAHs into more reactive carcinogens, such as benzo[a]pyrene-7,8-diol-9,10 epoxide (from benzo[a]pyrene), which is the most mutagenic and carcinogenic metabolite [4]–[6]. Thus, high EPHX1 enzymatic activity could increase the concentrations of carcinogens in the tissue. Hence, the *EPHX1* variants, individually or collectively with other metabolic enzymes, may lead to cancer susceptibility.

EPHX1 enzyme activity is affected by single or combination of polymorphisms Y113H and H139R [7], [8]. The present cancer meta-analysis was motivated by the idea that performing both single-SNP analysis and two-SNP analysis may provide insights into the relationship between EPHX1 enzyme activity and cancer risk. In single SNP analysis, we performed per-allele comparisons (H vs. Y; R vs. H) and pairwise comparisons (YH vs. YY, HH vs. YY; HR vs. HH, RR vs. HH) regardless of the inheritance model (dominant, co-dominant, recessive). Our combination analysis of two SNPs using the method of Benhamou et al. [9] assumed that the inheritance model was co-dominant, as the classification of low, intermediate and high activity was based on the counts of the high activity allele (H both for Y113H and H139R) of combination genotypes. We have additionally tested the inheritance model of these two polymorphisms by the Bayesian model-free approach [106], [107]. For lung cancer the results suggested a co-dominant inheritance model for the polymorphisms Y113H (λ = 0.64, 0.22–0.99) and H139R (λ = 0.62, 0.27–0.99). But in UADT this method suggested near dominant for Y113H (λ = 0.77, 0.34–1.00) and near recessive model for H139R (λ = 0.12, 0.08–0.90).

The xenobiotic metabolism of smoking products is carried out by both Phase I (e.g. cytochrome P450 family, EPHX1) and Phase II (e.g. glutathione-S-transferases) enzymes since Phase I enzymes induce the formation of active carcinogens from procarcinogens, whereas Phase II enzymes conjugate these compounds and make them suitable for excretion [108]. It is reasonable to think that the overall carcinogenic effect of tobacco compounds should be measured as the final result of the combined action of the two categories of enzymes. Further study is necessary to confirm the qualitative gene-gene interaction of these xenobiotic metabolism enzymes as well as their interaction with tobacco smoking dose in relation to susceptibility of tobacco-related cancers.

The dispersion extent of effect sizes or between-study heterogeneity in a meta-analysis determines the difficulty in drawing overall conclusions to a great extent [109]. Because the dispersion in observed effects is partly spurious (it includes both real difference in effects and also random error), before trying to interpret the variation in effects we need to determine what part of the observed variation is real. A critical meta-analysis should appropriately quantify the heterogeneity and thoroughly ascertain the caused reasons [110] such as using subgroup analysis, sensitive analysis and meta-regression. In the present study, both meta-regression and subgroup analysis by ethnicity revealed that ethnicity is a source of heterogeneity and have a major influence on the cancer risk of these two *EPHX1* polymorphisms. For instance, the Y113H low enzymatic activity allele (H) showed significant association with decreased risk of lung and UADT cancers in Caucasian (OR = 0.87, 95%CI = 0.81–0.94) but not significant in Asian (OR = 1.02, 95%CI = 0.89–1.16) (Table 2). The H139R high enzymatic activity allele (R) showed a more significant association with increased risk of cancer in both Asian (OR = 1.52, 95%CI = 1.13–2.05) and African (OR = 1.26, 95%CI = 1.01–1.57) than that in Caucasian (OR = 1.06, 95%CI = 0.97–1.15) (Table 2). The minor allele frequency of these polymorphisms in controls showed significant differences among different populations (Figure S1), which may have an impact on the statistical association analysis.

Study sample size was found to be the main source of heterogeneity for putative high vs. intermediate activity (Table S4) and the results are inconsistency when the studies were divided into subgroups of large sample size and small sample size (Table 3). Intuitively, larger samples studies should reach more convincing results. However, the large studies still exhibited as quite heterogeneous. Moreover, two large studies [40], [41] contributed mainly the between-study heterogeneity by applying method of Patsopoulos et al's sensitive analysis [23]. After omitted the heterogeneity-caused studies, putative high activity was significantly associated with increased risk of lung and UADT cancers (OR = 1.23, 95%CI = 1.06–1.42). Thus, putative high EPHX1 enzyme activity was supposed to increase risk lung and UADT cancers rather than decrease the risk as results from overall large sample subgroup analysis suggested.

Publication bias is another main limitation of meta-analysis which may arise from selective publication or selective inclusion of literatures. Obvious publication bias was detected from the analysis for R vs. H of H139R in pooled lung and UADT cancers. Studies of Zienolddiny [12], Ihsan [56], Graziano[44] and Wu a [37] were found remarkably deviated from other symmetrically distributed studies in the Begg's funnel plot (Figure S2). These four studies were exactly the source of heterogeneity by using Patsopoulos et al's sensitive analysis, which suggests that omitting heterogeneity-caused studies could reach relatively pertinent conclusions.

## Supporting Information

Table S1 Definition of EPHX1 activity predicted by single polymorphism Y113H/H139R and by combination of double polymorphisms.
(0.05 MB RTF)

Table S2 Characteristics of published studies included in the meta-analysis.
(0.73 MB RTF)

Table S3 Characteristics of the studies evaluated putative EPHX1 enzyme activity predicted by genotype combination of Y113H/H139R and cancer risk.
(0.17 MB RTF)

Table S4 Results of random-effect meta-regression for search of the source of heterogeneity.
(0.10 MB RTF)

Figure S1 Minor allele frequency of polymorphisms Y113H and H139R among ethnicity of African, Caucasian, East Asian and South Asian in controls.
(0.03 MB TIF)

Figure S2 Begg's funnel plot with pseudo 95% confidence limits for publication bias detection. Each point represents a separate study and is plotted by individual study log OR again the standard error of the log OR. A, H vs. Y of Y113H; B, R vs. H of H139R.
(0.06 MB TIF)

Text S1 Six case-control studies of EPHX1 polymorphisms and cancer risk were excluded for the following reasons.
(0.03 MB DOC)
