# Supplementary material for: Putative EPHX1 Enzyme Activity Is Related with Risk of Lung and Upper Aerodigestive Tract Cancers: A Comprehensive Meta-Analysis
Source: PLoS One. 2011 Mar 18;6(3):e14749. doi: 10.1371/journal.pone.0014749 (PMC3060809; doi:10.1371/journal.pone.0014749)
Supplement: Table S1 — Definition of EPHX1 activity predicted by single polymorphism Y113H/H139R and by combination of double polymorphisms. (0.05 MB RTF) [file pone.0014749.s001.rtf]

Table S1. Definition of EPHX1 activity predicted by single polymorphism Y113H/H139R and by combination of double polymorphisms
	Low activity	Intermediate activity	High activity	
Y113H	allele H, genotype HR and HH	allele Y, genotype YY		
H139R		allele H, genotype HH	allele R, genotype HR and RR	
Double polymorphisms	113HH/139HH, 113HH/139HR, 113YH/139HH	113HH/139RR, 113YY/139HH, 113YH/139HR	113YH/139RR, 113YY/139HR, 113YY/139RR	
