# Supplementary material for: Putative EPHX1 Enzyme Activity Is Related with Risk of Lung and Upper Aerodigestive Tract Cancers: A Comprehensive Meta-Analysis
Source: PLoS One. 2011 Mar 18;6(3):e14749. doi: 10.1371/journal.pone.0014749 (PMC3060809; doi:10.1371/journal.pone.0014749)
Supplement: Table S2 — Characteristics of published studies included in the meta-analysis. (0.73 MB RTF) [file pone.0014749.s002.rtf]

Table S2. Characteristics of published studies included in the meta-analysis.
Study 	year	Cancer types	Study 
design	SNPs studied	Country	Ethnicity	Genotyping	Cases/Controls
(Y113H; H139R)  	MAF in controls
(Y113H; H139R)	P for HWE
(Y113H; H139R)	
Lung cancer											
Smith [32]	1997	lung cancer	PCC	Y113H,H139R	UK	Caucasian	PCR-RFLP	50/203; 50/203	0.308; 0.145 	0.04; 0.48	
Benhamou [9]	1998	lung cancer	HCC	Y113H,H139R	France	Caucasian	PCR-RFLP	150/172; 150/172	0.404; 0.154 	0.36; 0.22	
Persson [33]	1999	lung cancer	PCC	Y113H	China	East Asian	PCR-RFLP	74/122	0.422	0.92	
London a [34]	2000	lung cancer	PCC	Y113H,H139R	USA	Caucasian	PCR-RFLP	182/458; 182/458	0.282; 0.192 	0.88; 0.35	
London b [34]	2000	lung cancer	PCC	Y113H,H139R	USA	African	PCR-RFLP	155/242; 155/242	0.209; 0.291 	0.57; 0.42	
Yoshikawa [35]	2000	lung cancer	PCC	Y113H,H139R	Japan	East Asian	PCR-RFLP	71/107; 71/107	0.435; 0.168 	0.76; 0.50	
To-Figueras [36]	2001	lung cancer	PCC	Y113H,H139R	Spain	Caucasian	AS-PCR/PCR-RFLP 	175/187; 175/187	0.307; 0.182 	0.36; 0.69	
Wu a [37]	2001	lung cancer	PCC	Y113H,H139R	USA	Caucasian	PCR-RFLP	51/64; 56/63	0.336; 0.062 	0.90; 0.14	
Wu b [37]	2001	lung cancer	PCC	Y113H,H139R	USA	African	PCR-RFLP	65/62; 75/71	0.226; 0.268 	0.54; 0.01	
Yin [38]	2001	lung cancer	HCC	Y113H,H139R	China	East Asian	PCR-RFLP	84/84; 84/84	0.440; 0.095 	0.31; 0.76	
Zhao[39]	2002	lung cancer	PCC	Y113H,H139R	USA	Caucasian	PCR-RFLP	162/153; 166/157	0.320; 0.185 	0.02; 0.73	
Cajas-Salazar [40]	2003	lung cancer	PCC	Y113H,H139R	USA	Caucasian	PCR-RFLP	110/119; 110/119	0.261; 0.176 	0.14; 0.28	
Gsur [10]	2003	lung cancer	HCC	Y113H,H139R	Austria	Caucasian	TaqMan	277/496; 277/496	0.329; 0.177 	0.93; 0.91	
Park [11]	2005	lung cancer	PCC	Y113H,H139R	USA	Mixed	SSCP/PCR-RFLP	178/365; 182/363	0.421; 0.193 	0; 0.40	
Voho [41]	2006	lung cancer	PCC	Y113H	Finland	Caucasian	PCR-RFLP /TaqMan	227/2083	0.298 	0.71	
McKay [42]	2008	lung cancer	HCC&PCC	Y113H,H139R	Six European countries	Caucasian	TaqMan	2155/2794; 2178/2800	0.331; 0.211	0.74; 0.25	
Zienolddiny [12]	2008	lung cancer	PCC	H139R	Norway	Caucasian	APEX	318/361	0.266 	0.08	
Rotunno [43]	2009	lung cancer	PCC	H139R	Italy	Caucasian	TaqMan	1858/2026	0.195 	0.52	
Grazianoa[44]	2009	lung cancer	NR	Y113H,H139R	Italy	Caucasian	Sequencing	42/72; 42/72	0.222;0.215	0.02; 0.64	
Timofeeva [45]	2010	lung cancer	PCC	Y113H,H139R	Germany	Caucasian	MALDI-TOF MS	611/1266; 613/1160	0.299; 0.194 	0.46; 0.83	
UADT cancers	
Jourenkova-Mironova [13]	2000	oral, pharynx, larynx cancer	HCC	Y113H,H139R	France	Caucasian	PCR-RFLP	250/172; 250/172	0.404; 0.154 	0.36; 0.22	
Amador [46]	2002	oral carcinoma	PCC	Y113H,H139R	USA 	Mixed	PCR-RFLP	137/99; 137/99	0.551; 0.157 	0.69; 0.05	
To-Figueras [47]	2002	larynx carcinoma	PCC	Y113H,H139R	Spain	Caucasian	PCR-RFLP	204/203; 204/203	0.310; 0.187 	0.24; 0.24	
Casson [48]	2003	esophageal adenocarcinoma	PCC	Y113H,H139R	Canada	Caucasian	PCR-RFLP	45/45; 45/49	0.311; 0.224 	0.80; 0	
Park a [14]	2003	oral cancer	HCC	Y113H,H139R	USA 	African	PCR-RFLP	81/122; 81/122	0.184; 0.303 	0; 0.74	
Park b [14]	2003	oral cancer	HCC	Y113H,H139R	USA 	Caucasian	PCR-RFLP	142/213; 142/213	0.376; 0.178 	0; 0.92	
Wenghoefer [49]	2003	oral, pharynx, larynx cancer	HCC	Y113H,H139R	Germany	Caucasian	AS-PCR 	280/289; 280/289	0.308; 0.215 	0.51; 0.13	
Zhang [50]	2003	esophageal carcinoma	PCC	Y113H	China	East Asian	PCR-RFLP	257/252	0.556 	0	
Casson [51]	2006	esophageal adenocarcinoma	HCC	Y113H,H139R	Canada	Caucasian	PCR-RFLP	56/95; 56/95	0.379; 0.247 	0.02; 0.51	
Lin [52]	2006	esophageal carcinoma	PCC	Y113H	Taiwan	East Asian	PCR-RFLP	145/352	0.497 	0	
Boccia [53]	2008	oral, pharynx, larynx cancer	HCC	Y113H,H139R	Italy	Caucasian	PCR-RFLP	210/241; 209/242	0.284; 0.207 	0.02; 0.09	
Lacko [54]	2008	oral, pharynx cancer	PCC	Y113H,H139R	Netherlands	Caucasian	PCR-RFLP	429/419; 429/419	0.311; 0.197 	0.55; 0.70	
McKay [42]	2008	oral, pharynx, larynx and esophagus cancer	HCC&PCC	Y113H,H139R	Six European countries	Caucasian	TaqMan	791/2519; 780/2527	0.332; 0.215	0.76; 0.18	
Varela-Lema [55]	2008	oral and pharynx cancer	HCC	H139R	USA	Caucasian	PCR-RFLP 	92/130	0.165 	0.10	
Ihsan[56]	2010	esophageal cancer	PCC	Y113H,H139R	India	South Asian	PCR-RFLP	142/185;142/185	0.443;0.132	0.48;0.43	
Soucek[57]	2010	oral, pharynx, larynx and cancer	PCC	Y113H,H139R	Czech and Poland	Caucasian	TaqMan	116/113;166/122	0.319;0.201	0.82;0.61	
Colorectal cancer	
Harrison [58]	1999	colon cancer	PCC	Y113H,H139R	Scotland	Caucasian	PCR-RFLP	101/203; 101/203	0.308; 0.145 	0.04; 0.47	
Sachse [15]	2002	colorectal cancer	PCC	Y113H,H139R	UK	Caucasian	PCR-RFLP	489/592; 490/593	0.381; 0.194 	0; 0.07	
Landi [59]	2005	colorectal cancer	PCC	Y113H,H139R	Spain	Caucasian	APEX	363/323; 361/321	0.285; 0.167	0.44; 0.40	
Robien [60] 	2005	colorectal cancer	PCC	Y113H,H139R	USA	Mixed	PCR-RFLP	1593/1960; 1593/1960	0.292; 0.199 	0.42; 0.15	
Tranah a [61]	2005	colorectal cancer	NCC	Y113H,H139R	USA	Caucasian	TaqMan	190/473; 189/466	0.318; 0.183 	0.69; 0.83	
Tranah b [61]	2005	colorectal cancer	NCC	Y113H,H139R	USA	Caucasian	TaqMan	253/418; 255/418	0.278; 0.194 	0.49; 0.83	
Van der Logt [62]	2006	colorectal cancer	PCC	Y113H,H139R	Netherlands	Caucasian	PCR-RFLP	365/391; 371/414	0.293; 0.196 	0.71; 0.72	
Kiss [63]	2007	colorectal cancer	PCC	Y113H,H139R	Hungary	Caucasian	PCR-RFLP	500/500; 500/500	0.283; 0.181 	0.05; 0.05	
Skjelbred [64]	2007	colorectal cancer	PCC	Y113H,H139R	Norway	Caucasian	PCR-RFLP	102/299; 101/299	0.334; 0.214 	0.91; 0.07	
Cotterchio [65]	2008	colorectal cancer	PCC	Y113H	Canada	Caucasian	TaqMan	832/1249	0.301 	0.98 	
Cleary[66]	2010	colorectal cancer	PCC	Y113H	Canada	Caucasian	TaqMan	1163/1292	0.304	0.87	
Hlavata[67]	2010	colorectal cancer	HCC	Y113H,H139R	Czech	Caucasian	TaqMan	495/495;495/495	0.319;0.229	0.75;0.31	
Colorectal adenoma	
Cortessis [68]	2001	colorectal adenoma	PCC	Y113H,H139R	USA 	Mixed 	PCR-RFLP	461/502; 461/501	0.383; 0.175 	0; 0.59	
Ulrich [69]	2001	colorectal adenoma	PCC	Y113H,H139R	USA 	Mixed	PCR-RFLP	693/600; 692/596	0.288; 0.201 	0.34; 0.77	
Tiemersma [70]	2004	colorectal adenoma	HCC	Y113H,H139R	Netherlands	Caucasian	PCR-RFLP	390/406; 389/407	0.317; 0.217 	0.09; 0.001	
Tranah a [71]	2004	colorectal adenoma	NCC	Y113H,H139R	USA 	Caucasian	TaqMan	525/522; 536/527	0.371; 0.200 	0; 0.56	
Tranah b [71]	2004	colorectal adenoma	NCC	Y113H,H139R	USA 	Caucasian	TaqMan	360/696; 365/708	0.326; 0.194 	0; 0.17	
Huang [16]	2005	colorectal adenoma	PCC	Y113H,H139R	USA 	Mixed	TaqMan	712/729; 702/724	0.309; 0.186 	0.08; 0.81	
Mitrou [72]	2007	colorectal adenoma	PCC	H139R	UK	Caucasian	PCR-RFLP	844/871	0.199 	0.45	
Skjelbred [64]	2007	colorectal adenoma	PCC	Y113H,H139R	Norway	Caucasian	PCR-RFLP	749/299; 749/299	0.334; 0.214 	0.91; 0.07	
Northwood [73]	2010	colorectal adenoma	PCC	Y113H,H139R	UK	Caucasian	TaqMan	312/303; 308/296	0.294; 0.236 	0.60; 0.89	
Breast cancer											
Sarmanova [74]	2004	breast cancer	HCC	Y113H,H139R	Czech	Caucasian	PCR-RFLP	237/311; 238/310	0.325; 0.234 	0.11; 0.54	
Spurdle [18]	2007	breast cancer	PCC	Y113H	Australia	Caucasian	TaqMan	1238/663	0.326 	0.01 	
Justenhoven [75]	2008	breast cancer	PCC	Y113H,H139R	Germany	Caucasian	MALDI-TOF MS	605/609; 601/624	0.295; 0.208 	0.12; 0.35	
Khedhaier [76]	2008	breast cancer	PCC	Y113H	Tunisia	Caucasian	PCR-RFLP	306/244	0.301 	0.06 	
Sangrajrang [77]	2009	breast cancer	PCC	Y113H,H139R	Thailand	South Asian	TaqMan	557/487; 562/489	0.490; 0.147 	0.74; 0.35	
MARIE-GENICA[78]	2009	breast cancer	PCC	Y113H,H139R	Germany	Caucasian	MALDI-TOF MS	3147/5483; 3142/5476	0.301;0.2	0.65;0.03	
Bladder and urinary tract cancers	
Brockmoller [79]	1996	bladder cancer	HCC	Y113H,H139R	Germany	Caucasian	PCR-RFLP	372/336; 360/330	0.264; 0.209 	0.01; 0.39	
Broberg [80]	2005	bladder cancer	PCC	Y113H,H139R	Sweden	Caucasian	TaqMan	61/154; 61/155	0.289; 0.248 	0.22; 0.13	
Figueroa [81]	2008	bladder cancer	HCC	Y113H,H139R	Spain 	Caucasian	TaqMan	1075/1009; 1087/1011	0.261; 0.191 	0.04; 0.97	
Hsu [82]	2008	urinary tract cancer	HCC	Y113H	Taiwan	East Asian	PCR-RFLP	196/210	0.400 	0 	
Srivastava [17]	2008	bladder cancer	HCC	Y113H,H139R	India	South Asian	PCR-RFLP	106/160; 106/160	0.313; 0.253 	0.38; 0.25	
Blood cancers											
Sarmanova [83]	2001	lymphoma	PCC	Y113H,H139R	Czech	Caucasian	PCR-RFLP	221/447; 224/453	0.375; 0.205 	0.01; 0.26	
Lebailly a [84]	2002	leukemia	PCC	Y113H,H139R	UK	Caucasian	PCR-RFLP	43/71; 43/71	0.387; 0.225 	0.01; 0.68	
Lebailly b [84]	2002	leukemia	PCC	Y113H,H139R	UK	Caucasian	PCR-RFLP	48/83; 49/83	0.361; 0.145 	0.31; 0.12	
Clavel [85]	2005	leukemia	HCC	Y113H,H139R	France	Caucasian	PCR-RFLP	218/105; 219/105	0.348; 0.214 	0.06; 0.10	
De Roos [86]	2006	lymphoma	PCC	Y113H,H139R	USA 	Caucasian	TaqMan	1103/921; 1091/916	0.283; 0.211 	0.45; 0.95	
Lincz [87]	2007	multiple myeloma	PCC	Y113H,H139R	UK	Caucasian	PCR-RFLP	91/199; 88/201	0.309; 0.167 	0.51; 0.19	
Gold a [88]	2009	multiple myeloma	PCC	H139R	USA 	African 	TaqMan	38/54	0.315 	0.68	
Gold b [88]	2009	multiple myeloma	PCC	H139R	USA 	Caucasian	TaqMan	222/682	0.210 	1.00	
Silveira [89]	2009	leukemia	HCC	H139R	Brazil	Mixed	PCR-RFLP	123/300	0.172 	0.09	
Chauhan[90]	2010	leukemia	PCC	Y113H,H139R	India	South Asian	PCR-RFLP	120/202; 120/202	0.408; 0.238	0.07; 0.35	
Liver cancer											
Wong [91]	2000	hepatocellular carcinoma	PCC	Y113H,H139R	UK	Caucasian	PCR-RFLP	46/203; 39/203	0.308; 0.145 	0.04; 0.47	
Tiemersma [92]	2001	hepatocellular carcinoma	PCC	Y113H,H139R	Sudan	African 	PCR-RFLP	110/193; 110/184	0.207; 0.258 	0; 0.77	
Kirk [93]	2005	hepatocellular carcinoma	HCC	Y113H	Gambia	African 	PCR-RFLP	195/351	0.161 	0	
Kiran[94]	2008	hepatocellular carcinoma	NR	Y113H,H139R	India	South Asian	PCR-RFLP	149/294;63/169	0.509;0.423	0.59;0.58	
Other cancers											
De Roos [95]	2006	brain cancer	HCC	Y113H	USA 	Mixed	TaqMan	595/527	0.287 	0.95 	
Sierra-Torres [96]	2003	cervical cancer	PCC	Y113H	USA 	Mixed	PCR-RFLP	69/72	0.125 	0.35 	
Nishino [97]	2008	cervical cancer	HCC	Y113H,H139R	Japan 	East Asian	TaqMan	124/117; 124/117	0.244; 0.179 	0; 0	
Agudo[98]	2006	gastric cancer	NCC	Y113H,H139R	Ten European countries	Caucasian	HRM	243/937; 242/944	0.291; 0.195 	0.01; 0.41	
Boccia [99]	2007	gastric cancer	HCC	Y113H,H139R	Italy	Caucasian	PCR-RFLP	106/250; 105/254	0.292; 0.211 	0.04; 0.05	
Spurdle [100]	2001	ovarian cancer	PCC	Y113H	Australia	Caucasian	TaqMan	545/287	0.307 	0.26 	
Baxter [101]	2002	ovarian cancer	PCC	Y113H,H139R	UK	Caucasian	PCR-RFLP	291/257; 291/257	0.304; 0.230 	0.20; 0.85	
Ockenga [102]	2009	pancreatic adenocarcinoma	PCC	Y113H	Germany and Netherlands	Caucasian	HRM	367/679	0.323	0.22	
Mittal [103]	2007	prostate cancer	NR	Y113H,H139R	India	South Asian	PCR-RFLP	130/140; 130/140	0.343; 0.268 	0.56; 0.20	

HCC, hospital-based case-control study; PCC, population-based case-control study; NCC, nested case-control study; RFLP, restriction fragment length polymorphism; APEX, arrayed primer extension; AS-PCR, allele-speciﬁc polymerase chain reaction; HRM, High Resolution Melting; MALDI-TOF MS, matrix assisted laser desorption ionisation time-of-flight mass spectrometry; MAF, minor allele frequency; HWE, Hardy-Weinberg Equilibrium; NR, not reported
