# Supplementary material for: Putative EPHX1 Enzyme Activity Is Related with Risk of Lung and Upper Aerodigestive Tract Cancers: A Comprehensive Meta-Analysis
Source: PLoS One. 2011 Mar 18;6(3):e14749. doi: 10.1371/journal.pone.0014749 (PMC3060809; doi:10.1371/journal.pone.0014749)
Supplement: Table S3 — Characteristics of the studies evaluated putative EPHX1 enzyme activity predicted by genotype combination of Y113H/H139R and cancer risk. (0.17 MB RTF) [file pone.0014749.s003.rtf]

Table S3. Characteristics of the studies evaluated putative EPHX1 enzyme activity predicted by genotype combination of Y113H/H139R and cancer risk
First author and 	Cancer 	Country	Ethnicity	Cases/	Cases (%)		Controls (%)	
year	type			Controls	L	I	H		L	I	H	
Benhamou, 1998 [9]	Lung cancer	France	Caucasian	150/172	50(33.3)	63(42.0)	37(24.7)		85(49.4)	65(37.8)	22(12.8)	
London a, 2000 [34]	Lung cancer	USA	Caucasian	182/458	67(36.8)	88(48.4)	27(14.8)		165(36.0)	194(42.4)	99(21.6)	
London b, 2000 [34]	Lung cancer	USA	African	155/242	26(16.8)	65(41.9)	64(41.3)		54(22.3)	101(41.7)	87(36.0)	
To-Figueras, 2001 [36]	Lung cancer	Spain	Caucasian	175/187	55(31.4)	87(49.7)	33(18.9)		70(37.4)	88(47.1)	29(15.5)	
Zhao, 2002 [39]	Lung cancer	USA	Caucasian	148/147	43(29.1)	65(43.9)	40(27.0)		47(32.0)	74(50.3)	26(17.7)	
Cajas-Salazar, 2003 [40]	Lung cancer	USA	Caucasian	110/119	30(27.3)	48(43.6)	32(29.1)		34(28.6)	70(58.8)	15(12.6)	
Park, 2005[11]	Lung cancer	USA	Caucasian	178/363	25(14.0)	117(65.7)	36(20.2)		80(22.0)	232(63.9)	51(14.0)	
Voho, 2006[41]	Lung cancer	Finland	Caucasian	227/2077	72 (31.7)	132 (58.1)	23 (10.2)		733 (35.3)	1047 (50.4)	297 (14.3)	
Jourenkova-Mironova, 2000 [13]	UADT (oral, pharynx, larynx)	France	Caucasian	250/172	85(34.0)	114(45.6)	51(20.4)		85(49.5)	65(37.8)	22(12.8)	
To-Figueras, 2002 [47]	UADT (larynx) 	Spain	Caucasian	204/203	76(37.3)	91(44.6)	37(18.1)		76(37.4)	94(46.3)	33(16.3)	
Casson, 2003 [48]	UADT (esophagus)	Canada	Caucasian	45/45	17(37.8)	16(35.6)	12(26.7)		19(42.2)	20(44.4)	6(13.3)	
Wenghoefer, 2003 [49]	UADT (oral, pharynx, larynx)	German	Caucasian	280/289	90(32.1)	135(48.2)	55(19.6)		104(36.0)	124(42.9)	61(21.1)	
Park a, 2003 [14]	UADT (oral)	USA	Caucasian	142/213	52(36.6)	51(35.9)	39(27.5)		85(39.9)	93(43.7)	35(16.4)	
Park b, 2003 [14]	UADT (oral)	USA	African	81/122	15(18.5)	27(33.3)	39(48.1)		24(19.7)	48(39.3)	50(41.0)	
Casson, 2006 [51]	UADT (esophagus)	Canada	Caucasian	56/95	15(26.8)	27(48,2)	14(25.0)		37(38.9)	38(40.0)	20(21.1)	
Lacko, 2008 [54] 	UADT (oral, pharynx, larynx)	Netherlands	Caucasian	429/419	158(36.8)	192(44.8)	79(18.4)		158(37.7)	184(43.9)	77(18.4)	
Soucek, 2010[57]		UADT (oral, pharynx, larynx)	Czech and Poland	Caucasian	116/113	38(32.8)	55(47.4)	23(19.8)		44(38.9)	49(43.4)	20(17.7)	
Cortessis, 2001 [68]	Colorectal adenomas	USA	Mixed	460/498	189(41.1)	175(38.0)	96(20.9)		227(45.6)	182(36.5)	89(17.9)	
Sachse, 2002 [15]	Colorectal cancer	UK	Caucasian	489/592	197(40.3)	208(42.5)	84(17.2)		246(41.6)	240(40.5)	106(17.9)	
Huang, 2005 [16]	Colorectal adenomas	USA	Mixed	772/777	245(31.7)	285(36.9)	242(31.3)		263(33.8)	317(40.8)	197(25.4)	
Robien, 2005[60]	Colon cancer	USA	Mixed 	1593/1960	306(19.2)	698(43.8)	589(37.0)		391(19.9)	889(45.4)	680(34.7)	
Lebailly a, 2002 [84]	Leukemia	UK	Caucasian	43/70	16(37.2)	17(39.5)	10(23.3)		25(35.7)	33(47.1)	12(17.1)	
Lebailly b, 2002 [84]	Leukemia	UK	Caucasian	48/83	20(41.7)	17(35.4)	11(22.9)		35(42.2)	38(45.8)	10(12.0)	
Lincz, 2007 [87]	Multiple myeloma	UK	Caucasian	85/100	28(32.9)	36(42.4)	21(24.7)		39(39.0)	45(45.0)	16(16.0)	
UADT, upper aerodigestive tract; L, low activity; I, intermediate activity; H, high activity
