# Supplementary material for: Putative EPHX1 Enzyme Activity Is Related with Risk of Lung and Upper Aerodigestive Tract Cancers: A Comprehensive Meta-Analysis
Source: PLoS One. 2011 Mar 18;6(3):e14749. doi: 10.1371/journal.pone.0014749 (PMC3060809; doi:10.1371/journal.pone.0014749)
Supplement: Table S4 — Results of random-effect meta-regression for search of the source of heterogeneity. (0.10 MB RTF) [file pone.0014749.s004.rtf]

Table S4. Results of random effect meta-regression for search of source of heterogeneity
Possible source of  
heterogeneity	113H vs. 113Y		139R vs. 139H		High vs. Intermediate	
	â coefficient (95%CI)	P-value	ô2		â coefficient (95%CI)	P-value	ô2		â coefficient (95%CI)	P-value	ô2	
Cancer type	0.02 (-0.15-0.18)	0.847	0.024		-0.10 (-0.29-0.09)	0.289	0.032		0.009 (-0.45-0.47)	0.969	0.11	
Ethnicity	-0.03 (-0.10-0.05)	0.489	0.022		0.12 (0.03-0.21)	0.010	0.021		0.04 (-0.65-0.73)	0.905	0.11	
Study design	0.01 (-0.10-0.12)	0.804	0.024		-0.01 (-0.14-0.12)	0.856	0.032		0.14 (-0.34-0.61)	0.552	0.10	
Sample size	-0.07 (-0.23-0.09)	0.296	0.022		0.17 (-0.01-0.35)	0.063	0.024		0.53 (0.19-0.88)	0.005	0.026	
Genotyping method	0.02 (-0.06-0.10)	0.590	0.023		0.04 (-0.03-1.12)	0.260	0.030		-0.10(-0.35-0.15)	0.427	0.10	
HWE-violation	0.01 (-0.17-0.19)	0.903	0.023		0.03 (-0.39-0.45)	0.901	0.031		¨D	¨D	¨D	
Cancer type: lung cancer or UADT; Ethnicity: Caucasian, Asian, African or Mixed population; Study design: hospital or population; Sample size : ¡Ý500 or <500; violating HWE: violated or not violated
