# Supplementary figures and images for: Putative EPHX1 Enzyme Activity Is Related with Risk of Lung and Upper Aerodigestive Tract Cancers: A Comprehensive Meta-Analysis
Source: PLoS One. 2011 Mar 18;6(3):e14749. doi: 10.1371/journal.pone.0014749 (PMC3060809; doi:10.1371/journal.pone.0014749)

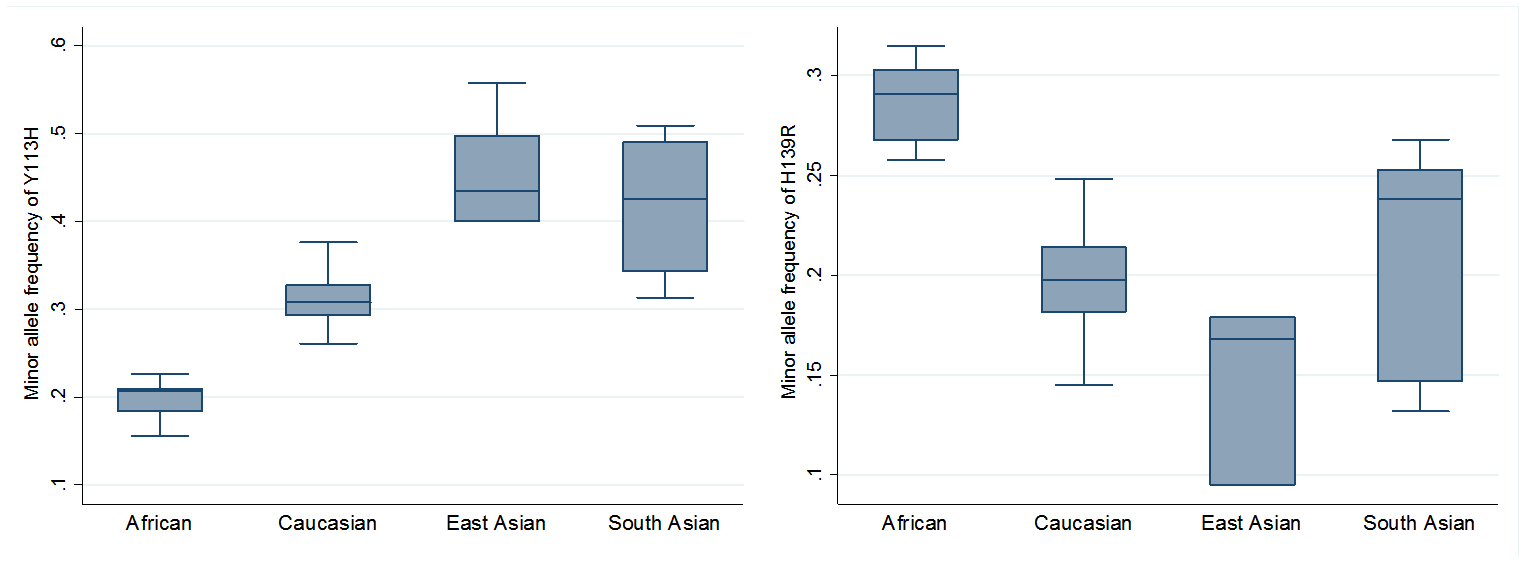

Supplement: Figure S1 — Minor allele frequency of polymorphisms Y113H and H139R among ethnicity of African, Caucasian, East Asian and South Asian in controls. (0.03 MB TIF) [file pone.0014749.s005.tif]
