# Supplementary material for: Putative EPHX1 Enzyme Activity Is Related with Risk of Lung and Upper Aerodigestive Tract Cancers: A Comprehensive Meta-Analysis
Source: PLoS One. 2011 Mar 18;6(3):e14749. doi: 10.1371/journal.pone.0014749 (PMC3060809; doi:10.1371/journal.pone.0014749)
Supplement: Text S1 — Six case-control studies of EPHX1 polymorphisms and cancer risk were excluded for the following reasons. (0.03 MB DOC) [file pone.0014749.s007.doc]

**Text S1.** Six case-control studies of *EPHX1* polymorphisms and cancer risk were excluded for the following reasons:

**Zhou, *et al.* 2001 [26]**, did not provide genotype data of EPHX1 polymorphisms.

**de Assis, *et al,* 2002 [27]**, did not provide complete genotype data of EPHX1 polymorphisms. The study merged genotype YH and HH of Y113H together. The same for polymorphism H139R, so we could not calculate the allele risk from this data.

**Tsai, *et al,* 2003[28]**, the reason is the same as that of de Assis, *et al*, 2002 [27].

**Gemignani, *et al*, 2007[29],** described results for the same source of samples with McKay, *et al,* 2008 [42].

**Rosenberger, *et al*, 2008[30],** described results for the same source of samples with Timofeeva, *et al,* 2010 [45].

**Kiran, *et al*. 2009 [31]**, the samples were just the same as that of Kiran *et al*. 2008 [94].
